# Electric-Field-Induced Amplitude Tuning of Ferromagnetic Resonance Peak in Nano-granular Film FeCoB-SiO_2_/PMN-PT Composites

**DOI:** 10.1186/s11671-016-1717-x

**Published:** 2016-11-08

**Authors:** Mei Luo, Peiheng Zhou, Yunfeng Liu, Xin Wang, Jianliang Xie

**Affiliations:** 1 National Engineering Research Center of Electromagnetic Radiation Control Materials, University of Electronic Science and Technology of China, Chengdu, 610054 China; 2 State Key Laboratory of Electronic Thin Films and Integrated Devices, University of Electronic Science and Technology of China, Chengdu, 610054 China

**Keywords:** Magnetoelectric effect, Nano-granular films, Ferromagnetic resonance, Magnetic anisotropy

## Abstract

One of the challenges in the design of microwave absorbers lies in tunable amplitude of dynamic permeability. In this work, we demonstrate that electric-field-induced magnetoelastic anisotropy in nano-granular film FeCoB-SiO_2_/PMN-PT (011) composites can be used to tune the amplitude of ferromagnetic resonance peak at room temperature. The FeCoB magnetic particles are separated from each other by SiO_2_ insulating matrix and present slightly different in-plane anisotropy fields. As a result, multi-resonances appear in the imaginary permeability (*μ*″) curve and mixed together to form a broadband absorption peak. The amplitude of the resonance peak could be modulated by external electric field from 118 to 266.

## Background

With the rapid development of electronic industries, the electromagnetic interference problem has become increasingly important and provides challenges for material researchers and radio frequency (RF) design engineers [1]. In order to reduce the undesirable electromagnetic radiation, nano-structural magnetic films have been extensively studied as one of the magnetic shielding materials. Nano-multilayer composites and nanocrystalline films exhibiting large complex permeability (real part *μ'* and imaginary part *μ″* larger than 100) in the gigahertz (GHz) range are reported recently [2, 3]. However, the relatively large complex permittivity (exceeds 10^4^) of these kinds of materials is difficult to satisfy the impedance-matching condition in the design method of electromagnetic wave absorption [4]. Magnetic granular films consisting of nanometer-sized ferromagnetic metallic particles (Fe, Co, Ni, and their alloys) randomly distributed in dielectric matrix (SiO_2_, ZnO, and ZrO_2_) are developed to solve the above problem [5–8]. These ferromagnetic insulators present high resistivity and hence relatively small permittivity. Nonetheless, the amplitude value of complex permeability cannot be freely tuned in the same sample. This kind of amplitude tuning has important meaning to further implement the active shielding. Therefore, exploiting new frameworks which can present different amplitude value of complex permeability according to the application requirement at room temperature are significant and challenging tasks.

The artificial two-phase systems consisting of ferromagnetic (FM) and ferroelectric (FE) materials have been extensively studied due to attractive potential applications. This heterostructure permits the control of magnetism with the converse magnetoelectric (ME) effect [9, 10]. However, the previous attention on ME effect mainly centered on the study of continuous ferromagnetic films and rarely concerned with ferromagnetic granular films. In this work, ferromagnetic metal-insulator (FeCoB-SiO_2_) granular film was fabricated on the (011)-oriented 0.71Pb(Mg_1/3_Nb_2/3_)O_3_-0.29PbTiO_3_ (PMN-PT) piezoelectric substrate. The amplitude of ferromagnetic resonance peak was tuned from 118 to 266 through strong ME coupling at room temperature. This framework provides a simple, active, and efficient way to balance permeability and permittivity of microwave-absorbing films.

## Methods

**Fig. 1:**
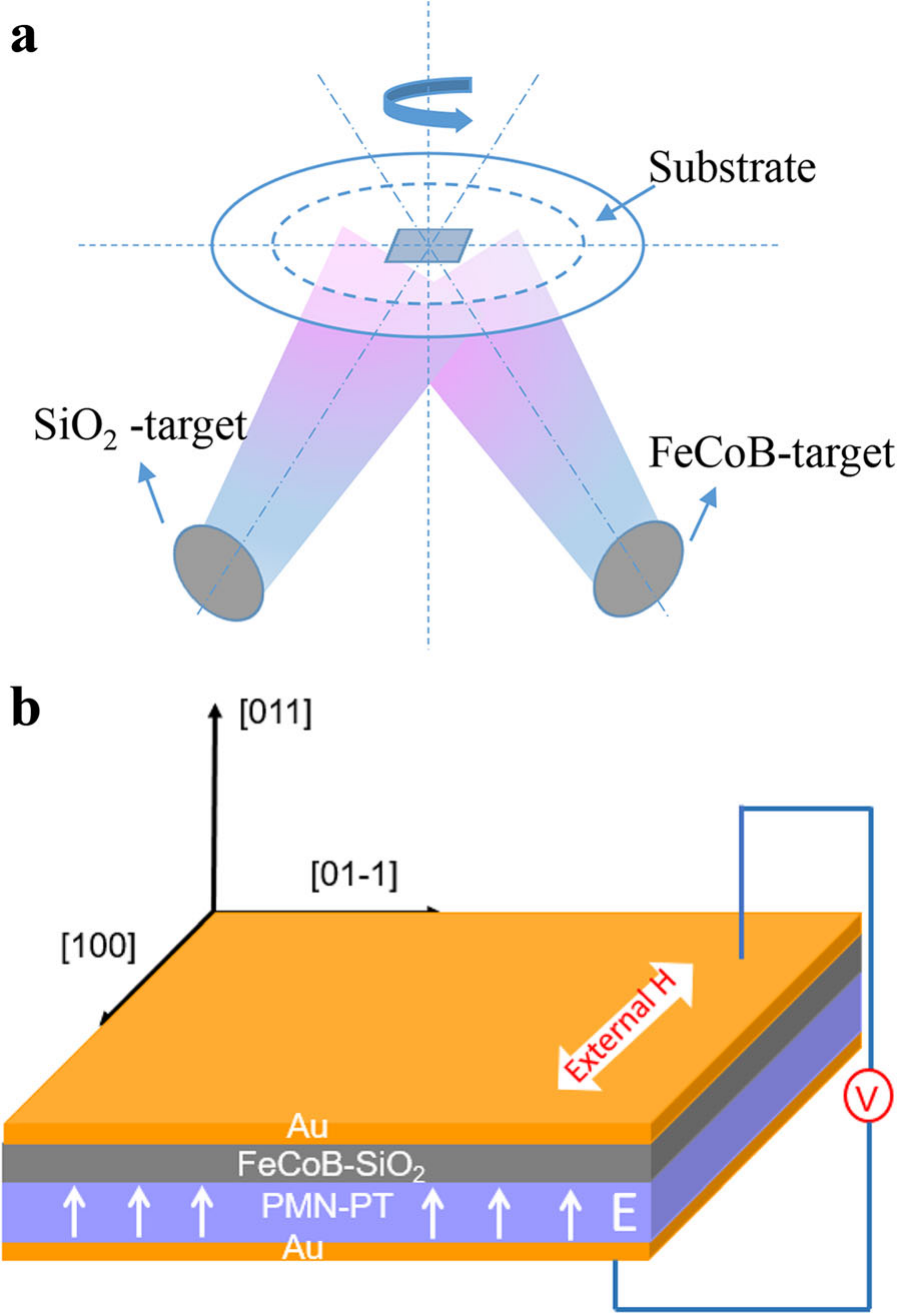
Schematic illustration of **a** sputtering system and **b** FeCoB-SiO2/PMN-PT heterostructure

FeCoB-SiO_2_ granular film was elaborated on the (011)-oriented PMN-PT substrate (10 mm × 5 mm × 0.5 mm) by magnetron co-sputtering system. The radio frequency magnetron sputtering was applied for SiO_2_ target with a power of 80 W, while DC magnetron sputtering was employed for (Fe_65_Co_35_)_90_B_10_ target with a power of 60 W. Figure 1a shows the schematic illustration of the sputtering system. The working pressure was 0.3 Pa, and the base pressure reached 4 × 10^−4^ Pa. An external magnetic field (H-field) about 200 Oe was applied along the [100] direction to introduce the in-plane uniaxial magnetic anisotropy in the film. Following the schematic illustration of FeCoB-SiO_2_/PMN-PT heterostructure shown in Fig. 1b, Au films were deposited on the top of FeCoB-SiO_2_ granular film and bottom of (011)-oriented PMN-PT substrate as electrodes.

The structure of granular film was investigated by X-ray diffraction (XRD, SHIMADZU XRD-7000) and transmission electron microscopy (TEM, JEM2100F). Hysteresis loops at different electric fields (E-fields) were measured by vibrating sample magnetometer (VSM, RIKEN, JAPAN). The microwave complex permeability was measured by a vector network analyzer (Agilent 8720ET) with the shorted microstrip method without bias H-field. The surface resistivity was measured by four point probe meter (SX1944) at room temperature.

## Results and Discussion

**Fig. 2:**
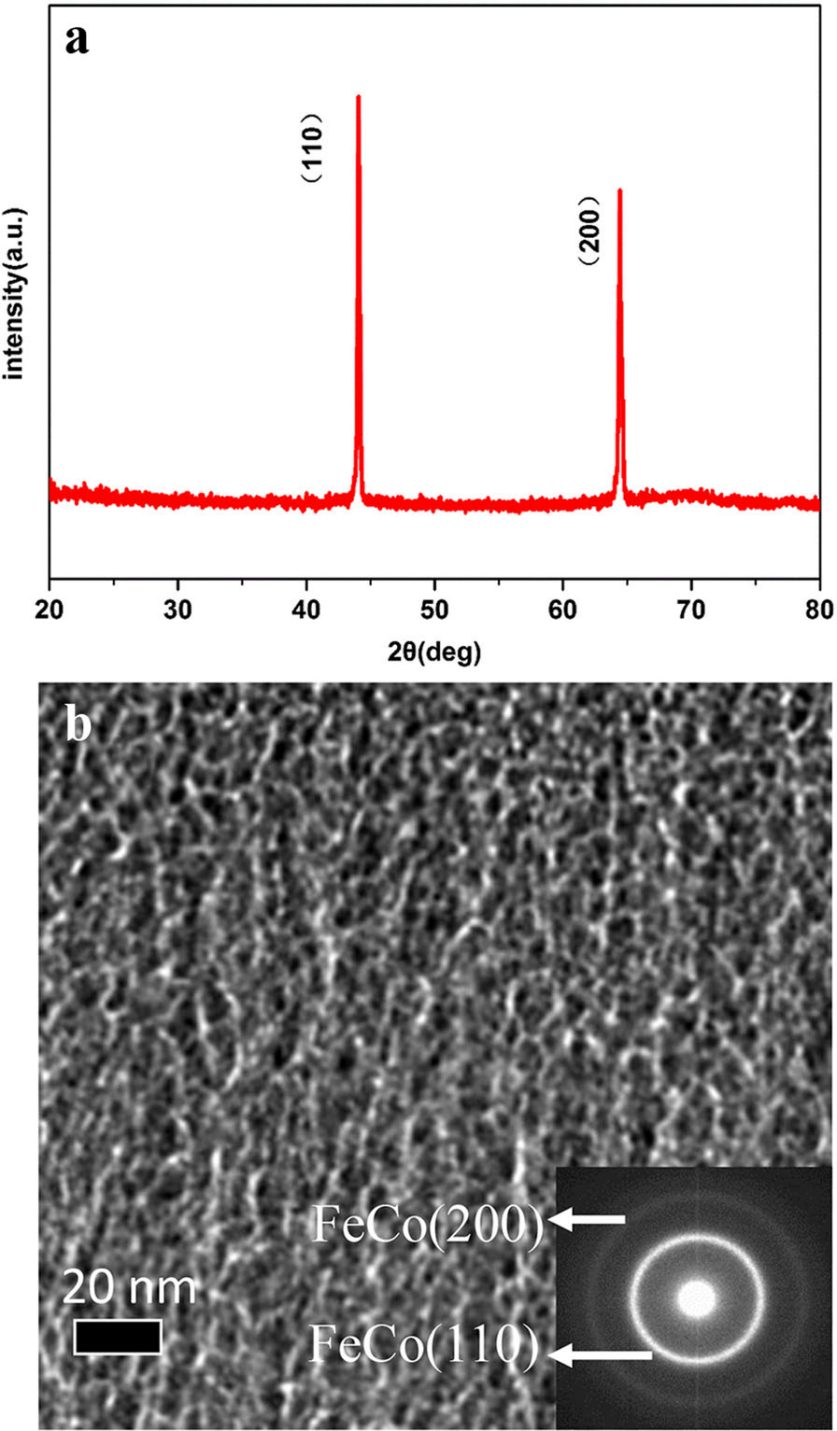
**a** X-ray diffraction pattern, **b** TEM image, and electron diffraction pattern of FeCoB-SiO_2_ granular film

The XRD diffraction pattern of FeCoB-SiO_2_ granular film is shown in Fig. 2a. The FeCoB phase presents polycrystalline structure according to the appearance of (110)_FeCo_ and (200)_FeCo_ diffraction peaks. Diffraction peaks of SiO_2_ phase are not observed, which indicates that the SiO_2_ is an amorphous phase. Figure 2b shows the TEM image and electron diffraction pattern of the FeCoB-SiO_2_ granular film. It is found that SiO_2_ phase (shows in bright contrast) wraps around the FeCoB alloy particles (show in dark contrast) and hence forms the insulating network matrix. The FeCoB particles have a non-uniform size distribution from 2 to 10 nm in diameter, and the distance between them are about 2 nm. The electron diffraction pattern from (110) and (200) planes indicates FeCoB particles are polycrystalline structure, which is consistent with the result of XRD measurement. The surface resistivity *ρ* of FeCoB-SiO_2_ granular film is 8550 μΩ cm, which is bigger than continuous FeCoB films (*ρ* is often given as close to zero).

**Fig. 3:**
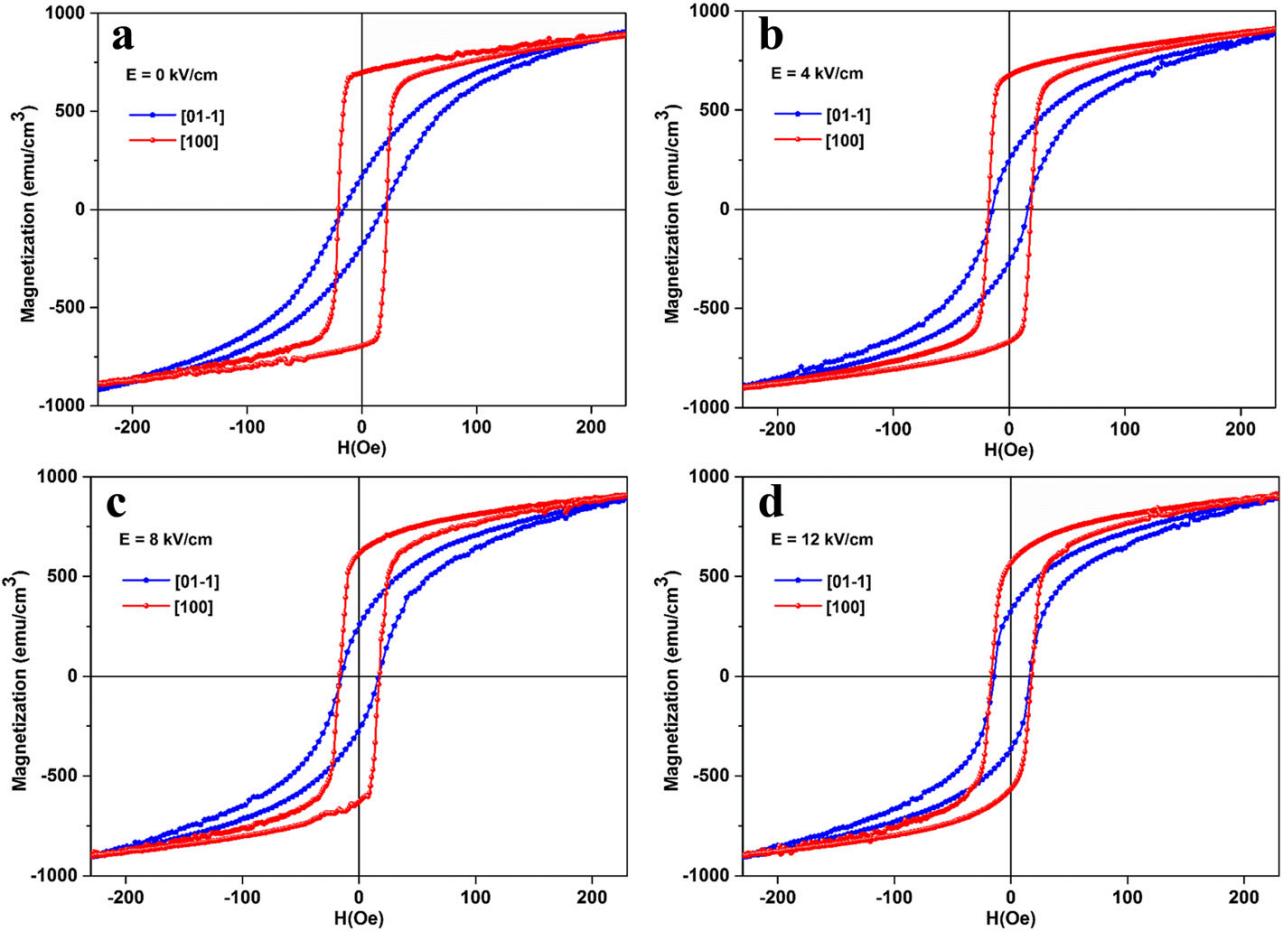
In-plane hysteresis loops of FeCoB-SiO_2_/PMN-PT at **a** 0 kV/cm, **b** 4 kV/cm, **c** 8 kV/cm, and **d** 12 kV/cm

Figure 3 shows the external E-field dependence of magnetic hysteresis loops of FeCoB-SiO_2_/PMN-PT composite. As can be seen from Fig. 3a, FeCoB-SiO_2_ granular film exhibits obvious in-plane uniaxial magnetic anisotropy with E-field of 0 kV/cm, where the curve obtained along the [01-1] direction shows hard axis behavior and the [100] direction shows easy axis behavior. When the E-field is applied through the thickness direction of PMN-PT substrate, the remanent magnetization of FeCoB-SiO_2_ granular film decreases along [100] direction and increase along [01-1] direction. This result implies a strong strain-driven ME coupling between FeCoB particles and PMN-PT substrate and a fundamental change of the magnetic anisotropy field in FeCoB-SiO_2_ granular film. A large E-field-induced magnetoelastic effective field (*H*_eff_) is expressed as [11]

1 $$ {H}_{\mathrm{eff}}=3\lambda YdE/{M}_s, $$

where *d* is the piezoelectric coefficient, *λ* is the magnetostriction constant, and *M*_*s*_ is the saturation magnetization. The *H*_eff_ coming from the ME coupling not only affects the static magnetic properties of FeCoB-SiO_2_ granular film but also affects the dynamic magnetic properties.

**Fig. 4:**
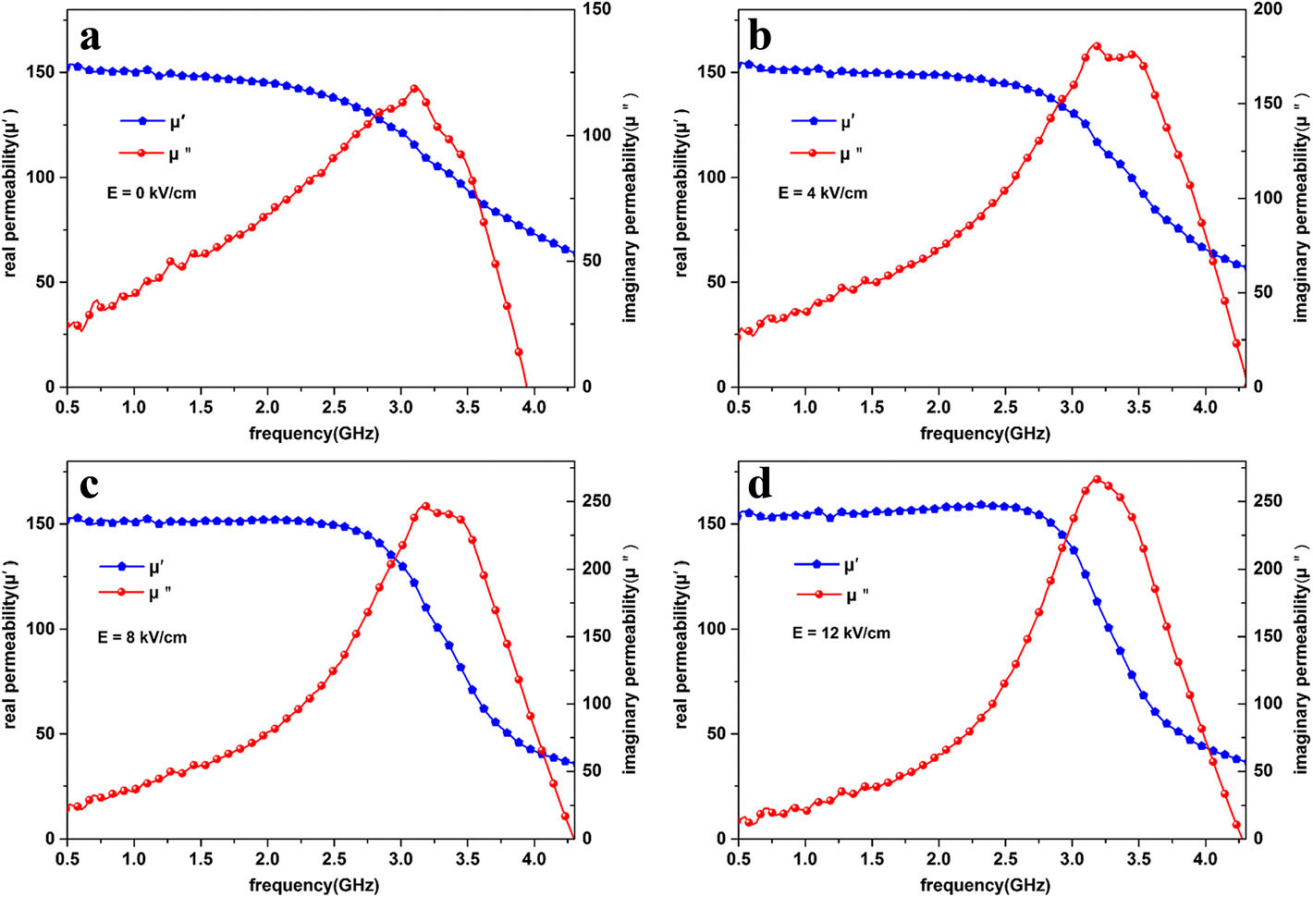
The E-field dependence of microwave complex permeability of FeCoB-SiO_2_/PMN-PT at **a** 0 kV/cm, **b** 4 kV/cm, **c** 8 kV/cm, and **d** 12 kV/cm

The complex permeability curves of the FeCoB-SiO_2_/PMN-PT composite under different E-fields across the thickness of the PMN-PT are shown in Fig. 4. For FeCoB-SiO_2_ granular film, it is considered that almost all the magnetic particles are separated from each other by non-magnetism phase because the FeCoB particles are embedded in SiO_2_ matrices as shown in Fig. 2b. Since the size of the magnetic particles is smaller than the critical value of single domain which is dozens of nanometers for Fe_65_Co_35_, FeCoB particles can be characterized by a single domain spontaneous state [12]. The ferromagnetic exchange interaction forces the magnetic moments to align parallel and deviates from the easy axis of each individual FeCoB particle. As a consequence, the local anisotropies of FeCoB-SiO_2_ granular film are average out according to the random anisotropy theory proposed by Herzer [13]. Thus, magnetic moment orientations of each single domain are along their respective preferred direction of magnetization and disordered in plane. In the meantime, the magnetic moments of interfacial atoms of magnetic particles are influenced by the pinning effect of the SiO_2_ on the interface. Each particle undergoes totally different pinning process due to the different shapes and sizes. Together with the abovementioned effects, it is considered that the in-plane anisotropy fields of each FeCoB particle are slightly different. These minor differences would not show up explicitly in static magnetic hysteresis loop, but implicitly as part of dynamic permeability spectra. As Fig. 4a shows, real permeability (*μ'*) curve exhibits slight step, which is similar to FeCoB/SiO_2_/FeCoSiB magnetic films. Owing to the differences between FeCoB and FeCoSiB magnetic properties, two resonance peaks of the complex permeability spectra are observed in [3]. In our case, the multiple magnetic states also result in multi-resonance phenomenon and broaden the resonance band by mixing all the resonance bands together. When the E-field is applied, the E-field-induced in-plane effective anisotropic field (*H*_eff_) influences the shift of ferromagnetic resonance frequency (*f*_r_) by the Kittel equation [14]

2 $$ {f}_r=\gamma \sqrt{\left({H}_r+{H}_{\mathrm{eff}}\right)\left({H}_r+{H}_{\mathrm{eff}}+4\pi {M}_s\right)}, $$

where *γ* is the gyromagnetic ratio. Based on the E-field dependence of static magnetic hysteresis loops, the total in-plane anisotropic fields of FeCoB-SiO_2_ granular film are decreased with E-field. The *H*_eff_ brings negative effect to total in-plane anisotropic fields. As a matter of fact, the strain responses of PMN-PT substrate under the converse piezoelectric effect are not homogeneous in plane [15]. This is negligible for continuous ferromagnetic films, but important for granular films. The strain of piezoelectric substrate reduces gradually from edge to center. When the E-field is 4 kV/cm, magnetic properties of nanoparticles which are located at the edge of substrate are effectively changed by *H*_eff_, while nanoparticles located at the center of substrate are basically unchanged. Hence, a part of ferromagnetic resonance peaks moves to low frequencies and mixes with those unchanged peaks located at low frequencies. Because of the additive effect of multiple ferromagnetic resonance peak, the amplitude of the peak has got an improvement. Along with the gradually increasing of E-field, magnetoelastic *H*_eff_ increases and affects more FeCoB particles. The amplitude of the ferromagnetic resonance peak is further increased due to the more peaks shift to low frequency and mix. We can see that multiple peak mixes into single peak and the amplitude of the resonance peak changes dramatically with E-fields, being 118 at 0 kV/cm, 181 at 4 kV/cm, 246 at 8 kV/cm, and 266 at 12 kV/cm, respectively. This framework realizes the tuning of amplitude value of the imaginary part *μ″* by E-fields in a certain frequency range.

## Conclusions

In summary, we have fabricated a heterostructure of FeCoB-SiO_2_/PMN-PT (011) and studied the ME effect by E-field-induced amplitude tuning of ferromagnetic resonance peak in GHz range. Almost all the FeCoB magnetic particles are separated from each other by non-magnetism phase SiO_2_ and the slightly different in the in-plane anisotropy fields. As a result, the imaginary permeability (*μ″*) curve presents broad-bandwidth and multi-peak resonance. The multiple resonant peaks mix into a single peak, and the amplitude of the resonance peak changes from 118 to 266 with increasing E-field. This framework provides an important opportunity for balancing permeability and permittivity of microwave absorbers.

## Abbreviations

E-field: Electric field
FE: Ferroelectric
FM: Ferromagnetic
*H*_eff_: Magnetoelastic effective field
ME: Magnetoelectric
PMN-PT: 0.71Pb(Mg_1/3_Nb_2/3_)O_3_-0.29PbTiO_3_
RF: Radio frequency
TEM: Transmission electron microscopy
VSM: Vibrating sample magnetometer
XRD: X-ray diffraction
